# Primary headaches during lifespan

**DOI:** 10.1186/s10194-019-0985-0

**Published:** 2019-04-08

**Authors:** Andreas Straube, Anna Andreou

**Affiliations:** 1 Department of Neurology, University Hospital LMU, Ludwig-Maximilians-University, 81377 Munich, Germany; 2 Headache Research, Wolfson CARD, Institute of Psychiatry, Psychology & Neuroscience, King’s College London, London, UK; 3 The Headache Centre, Guy’s and St Thomas’ NHS Foundation Trust, London, UK

**Keywords:** Migraine, Tension-type headache, Cluster headache, Clinical symptoms, Children, Adults, Elderly, Migraine-related syndromes, Aura, Sympathetic, Parasympathetic, Hypothalamus

## Abstract

Primary headaches are one of the most prevalent neurological disorders and can occur during a wide range of lifespan. Primary headaches, especially migraine, are cyclic disorders with a complex sequence of symptoms within every headache attack. There is no systematic review of whether these symptoms changes during lifespan. Indeed, the clinical presentation of migraine shows an age-dependent change with a significantly shorter duration of the attacks and occurrence of different paroxysmal symptoms, such as vomiting, abdominal pain or vertigo, in childhood and, in contrast, largely an absence of autonomic signs and a more often bilateral headache in the elderly. The age-dependent differences in the clinical presentation are less distinct in cluster headache and, especially, in tension-type headache. The differences in the clinical presentation are in agreement with the idea that the connectivity of hypothalamic areas with different brainstem areas, especially the central parasympathetic areas, is important for the clinical manifestation of migraine, as well as, the change during lifespan.

## Introduction

Pain, especially headache, is one of the most frequent complaints of patients seen in the outpatient clinic and in the emergency room. In order to diagnose the symptoms adequately, the changes in the symptomatology of pain disorders such as primary headaches during lifespan have to be taken in account. In general, pain perception changes with age and is different in very young and very old patients. In a systematic review of 12 studies, Tumi et al. [1] found that in the elderly subjects (mean age: 62 years) the pressure pain thresholds were lower than in the younger subjects (mean age: 22 years). Otherwise the heat pain thresholds did not differ. Furthermore, they also found evidence that younger children (6–8 years) were more sensitive to pain stimuli than older children (9–14 years). Another systematic review reports that the pain thresholds increase with age. Interestingly, the authors also found that these age-related changes were more accentuated in the trigeminal system [2]. Most of the published studies found also that pain symptoms are more prevalent in females than in males [3], although it is not always clear if this is related to a higher sensitivity across pain stimuli or to psychosocial factors such as a higher risk of catastrophizing [4]. Gonadal hormones may also play an important role. Estrogen may influence the descending pain inhibiting system and there is some evidence that estrogen antagonists may be helpful in chronic pain states [4]. In very young children and in newborns it is still not clear in which range they perceive pain [5]. On the other hand, the descending inhibitory spinothalamic fibers may not function at this age [5]. Concerning gender differences in children, Boerner et al. [6] stated that in the majority of studies there were no differences in the pain thresholds between girls and boys if the children were younger than 12 years. This finding may point towards a developmental factor in the sex differences in pain perception found later on. Experimental data concerning pain perception in the trigeminal area in very young children are missing.

## Background

Clinical presentation of primary headaches is age dependent.

It is obvious that such age- and sex-dependent differences in general pain perception should also influence the symptomatology of primary headache disorders. Indeed, there are some indications that the symptoms of primary headache syndromes change with age. Such differences have several implications for the clinical evaluation and the understanding of the underlying pathophysiology. In the acute situation it is important to notice that the pain perception and therefore also the by the patient described symptoms differ depending on age and gender.

1. Diagnostic criteria should reflect this by age-specific criteria and the clinical examiner should be aware of these age-dependent changes e.g. that in young children’s the headache is often not the leading symptom and that the attacks can be short as 30–60 min. Otherwise, in the elderly patient with a migraine in the history, the headache attacks become more like tension-type headache attacks and it is not clear if this reflects a change of the headache etiology or is based in the changed mechanism of the primary migraine.
2. Age-dependent differences result in the question of which neuronal structures and mechanisms are responsible for such a change in the clinical symptoms during lifespan.

The following overview will try to answer these questions and to give an overview about the so far published literature about the clinical variation of headache symptoms with age. In order to do so we concentrated us on primary headaches such as migraine, tension-type headache and cluster headache since data have been published only for these primary headaches. We will not focus on secondary headaches, which are important in the differential diagnosis of headaches in the very young, as well as, in the elderly. The basis for the reported literature was a search in the database of PubMed.

### Migraine

#### Prevalence

Migraine prevalence is strongly age-dependent. Most of the studies on headache prevalence in the very young did not differentiate between migraine and other primary headache due to the difficulties in classifying childhood headache. These difficulties are based in part on the limited language abilities in children under 6 to 7 years age. The 12-month migraine prevalence was 12.5% for 7–14-year-old children compared to 20.5% for older children in a questionnaire-based study in Hungarian schoolchildren [7]. In an epidemiological study in children younger than 5 years in Finland, headache was diagnosed in 19.5% of the children. No differentiation between migraine and tension-type headache was made, but there was a strong relationship between recurrent abdominal pain and frequent headache [8]. In a German study the 6-month prevalence for headache was 38.6% for the 7–8 year olds and 63.4% for the 13–14 year olds [9]. In a review of 37 studies on headache in children, Albers et al. found that the majority of these studies reported a significant increase of headache prevalence with age [10]. The migraine prevalence for younger children (below 7 years) does not differ between girls and boys [11, 12]. Studies focusing more on migraine also found an increase in prevalence with age (overview in: [10]). The prevalence of migraine varies, depending on the study and the age range of the included subjects, between 2.7% and 10.0% [12].

There are several studies about the prevalence of migraine in patients older than 18 years. In a telephone interview-based survey in the USA 145000 participants were screened. The migraine prevalence (fulfilling complete criteria) was highest for the age group 30 to 39 years (20.1%). The prevalence declined with age to a minimum of 3.9% (> 70 years) [13]. In a Swedish study the life-time prevalence was 31.5% and the 12-month prevalence 18.0% [14], which was slightly higher than the 6-month prevalence in the German study with about 11% for migraine and probable migraine [15].

In the same study, the 6-month prevalence in the 65–75-year-old group was about 3.5% for migraine and about 12.5% for tension-type headache, with females affected 2 to 1.5 times more often [15], and in a study from northern Italy the prevalence of migraine after the 75th year was 2.7% for males and 7.6% for females [16]. Concerning the first manifestation of migraine, it is not so rare that the first migraine attack occurs after the 50th year of life [17]. Otherwise first manifestation after the 60th year is very rare [18].

Concerning the severity of the primary headache patients over 70 years old with migraine, about 41% report on headache on 10–14 days per month [13, 14] and the average age of patients with chronic migraine is higher than that of patients with episodic migraine [19].

Migraine prevalence during lifespan is also gender-dependent [20]. Before puberty, migraine is slightly more common in boys, with the highest incidence between 6 to 10 years of age [20], but this is reversed with the onset of females’ cyclic hormonal changes [21, 22]. In general, women are more commonly affected than men with a lifetime prevalence of 12–17% and 4–6%, respectively [20, 23]. The male-to-female ratio in Europe is similar to that in America and Africa, although the exact percentages differ [24]. Estrogen withdrawal as seen in menstrual migraine is a reliable trigger of menstrual attacks in women and this may account for the increased prevalence of migraine in women compared to men in the reproductive years [25]. Hormonal changes in the perimenopause phase mark a period of increased migraine prevalence in women, during which maintenance of a stable estrogen environment with hormonal replacement therapy can have a positive effect on estrogen-withdrawal migraine [26]. Migraine in women usually declines after menopause [27, 28], further indicating the influence of hormonal changes on migraine occurrence.

#### Clinical symptoms in children

There is agreement that the clinical manifestation of migraine in childhood is different from that in adulthood. It is not clear if classical headache syndromes can occur in infancy, but migraine is diagnosed at the latest after the development of speech and one problem of finding the diagnosis may be related to the reduced lingual capabilities in young infants. The most obvious difference is that the headache attacks in children are much shorter than in adults. In a recent expert review the authors propose as a minimal attack duration, in contrast to the current IHCD criteria, 30 min which is much shorter than the minimum of 4 h for adult migraine and the 2 h for children [11, 29]. Other differences are that the pain is less often unilateral and phonophobia is relatively rarely reported [30]. Often the symptoms are quite unspecific, such as a paroxysmal episode of feeling unwell without any other reasons for it and some intolerance to light or reduced appetite [31]. Premonitory symptoms may occur even in very young children (up 18 months) and can be seen in 67%, which is slightly less than the percentage in adolescents (85%) [32]. Premonitory symptoms can be also fatigue, hunger, paleness, vertigo and dizziness, By comparing patients younger than 6 years with patients aged 6 to 12 years and 12 to 18 years, Eidlitz-Markus and colleagues found that there was no difference in the rates of unilateral headache, phono−/photophobia, nausea or worsening of the pain during physical activity between these groups [33]. But the duration of the attacks was shorter in the younger group and also vomiting was significantly more frequent in the youngest group [33]. Cranial autonomic signs are often seen in pediatric migraine [34]. Red ear syndrome, a uni- or bilateral hyperemia of the ear concha lasting for about 1 hour, was seen in up to 23% of children with migraine and was highly specific for migraine [35]. Another feature of childhood migraine is that there are a number of so-called migraine-related syndromes which rarely occur in adolescents or adults. There is clear scientific proof that a high proportion of these patients develop a clear migraine in later life [36]; any episodic syndrome in childhood increases the risk of later development of migraine by more than 50% compared to children without such a history of an episodic syndrome. The classical episodic syndromes related to migraine are benign paroxysmal torticollis, cyclic vomiting syndrome, abdominal migraine, and acute confusional migraine (Table 1). Brainstem and/or hypothalamic disturbances have been suggested as the underlying pathophysiology for at least some of these syndromes but no clear pathophysiological concept exists [37–39]. Interestingly, benign paroxysmal vertigo, which is commonly considered a migraine precursor, constitutes an age-specific manifestation of defective neuronal calcium channel activity, as it is frequently associated with mutations of the calcium channel *CACNA1A* gene [40–42], which is also involved in familial hemiplegic migraine [43]. There is also accumulating evidence that non rapid eye movement sleep disorders, such as sleep terrors, which involve hypothalamic centers [44], are related to migraine [36, 45]. The common factor is that otherwise healthy children develop paroxysmally clinical symptoms which resolve after some minutes to 48 h. The prevalence of these syndromes is low; some authors stated that 10% of childhood migraine cases also show such syndromes [46]. In a retrospective analysis of data from a large health insurance provider we found period prevalence of these syndromes of only about 3% [36].

**Table 1:** Migraine-related syndromes (modified from [35, 45])

Syndrome	Main symptom	Clinical signs	Duration	Prevalence
Abdominal migraine	Repeated attacks with midline abdominal pain	Accompanied by anorexia, nausea, vomiting, pallor	2–72 h	0.01%
Benign paroxysmal torticollis	Paroxysmal head tilt, sometimes also head rotation	Accompanied by pallor, irritability, malaise, vomiting, ataxia	Minutes to days	0.83%
Benign paroxysmal vertigo	Acute vertigo	Accompanied by nystagmus, unsteady gait, pallor, vomiting	Minutes to hours	0.43%
cyclic vomiting	attacks with intense nausea and vomiting, occurring periodically	Nausea/vomiting several times per hour	1 h to days	0.99%
Confusional migraine	Attacks with acute confusion	Restlessness, agitation, altered sensorium, disorientation, bizarre behavior	Less than 6 h	10% of childhood migraine
Somnambulism	Unclear if related, rising up from sleep and performing complex motor behavior	Typically occurring during slow-wave sleep, restless leg syndrome may be associated	Less than 1 h	0.07%

In summary, migraine attacks in children are significantly shorter and less specific than in adulthood, vegetative signs are more frequent and otherwise there are several paroxysmal syndromes, which are related to migraine which all show an activation/disinhibition of the brainstem and potentially hypothalamic structures in some aspects.

#### Clinical symptoms in adults

Premonitory symptoms, which occur prior to the actual migraine headache, are regularly reported by about 70% of migraine patients [46]. The most frequently reported symptoms are tiredness and disturbed concentration; other typical premonitory symptoms are nausea, food cravings, and yawning [46]. Increasing data from brain imaging studies in spontaneous or nitroglycerine-induced attacks suggest a role for the hypothalamus during this phase [47–50]. By which specific hypothalamic nuclei and which pharmacological alterations such symptoms, and potentially a migraine attack, are triggered remains however unknown. The occipital cortex, particularly the visual cortex, also appears to be activated during this premonitory phase; however, its role has not been investigated further [51].

During the headache phase, based on the findings of a large epidemiological study in the USA with more than 145,000 participants, about 80% of adult migraine sufferers report throbbing pain and more than 60% unilateral headache, which is classified as severe by nearly 80%. Physical activity worsens the headache in about 60% of 18–59-year-old patients. Associated symptoms such as nausea occur in more than 50%, photophobia and phonophobia in about 75%. Symptoms of an aura are described increasingly more often with increasing age: 13% in the 18–29-year-olds, and up to 25% for the 50–59-year-old patients [13]. In a subgroup of patients, vertigo and dizziness are also a major part of the migraine attack. Vestibular migraine mainly manifests in the age range from 8 to 53 years with a median of 23 years [52]. In the same study the authors found that the proportion of patients with a high-frequency migraine increased slightly with age from 12.5% for the 18–29-year-old patients to 24.5% for the 50–59-year-olds [13]. But it is important to bear in mind that migraine prevalence also decreases with age, thus the absolute number of patients with high-frequency migraine is still higher in younger adults. A Swedish study focusing on female patients did not find any association of migraine frequency or duration with age [14]. Rarer clinical characteristics can be also reported by patients, such as the Alice in Wonderland syndrome, which appears to be more prevalent in patients with vestibular migraine [53].

A number of studies investigated neural mechanisms involved in the development of the different headache symptoms. During the actual headache of a migraine attack, brain imaging studies found increased blood flow changes in the brainstem and pons area, in the thalamus, basal ganglia, and cortex [54–56]. In patients with nausea, a PET imaging study confirmed changes in the brain circuits mediating nausea, including the nucleus tractus solitarius, dorsal motor nucleus of the vagal nerve, and the nucleus ambiguus, as well as in the periaqueductal grey [57]. Although thalamic activation was initially considered to be part of the ascending trigemino-thalamic activation, its role in migraine deserves greater attention. Studies by Noseda and colleagues identified a unique retino-thalamic pathway that becomes activated by light and can modulate third-order trigemino-thalamic neurons [58, 59]. Sensitization of third-order thalamic neurons has also been identified as being involved in the development of allodynia during the headache phase of migraine [60, 61], a symptom that occurs in about 65% of patients [62]. Allodynia may also involve areas outside the trigeminal innervation which can only be explained by involvement of third-order thalamic neurons.

Migraine aura is believed to be the phenomenon of cortical spreading depression (CSD), which involves blood flow changes and neuronal and glial activation. It most likely occurs primarily in the visual cortex in migraine with aura patients [63, 64]. A number of studies in in migraine with aura patients in the interictal phase, have also demonstrated altered neuronal activity in the occipital cortex [65–68], as well as, in the motor cortex [69, 70], potentially due to disruption in GABAergic and glutamatergic transmission [67]. A thicker cortical cortex has been also reported in migraine with aura patients [71]. Although cortical hyperexcitability has been proposed in migraine without aura patients, meta-analysis studies suggest there is not enough evidence to support this hypothesis [72, 73]. Mathematical models show that probably different to CSD induction in rodents, CSD in humans can be locally circumscribed which may be explain why quite focal and specific symptoms like “Alice in Wonderland syndrome” can occur [74].

#### Clinical symptoms in the elderly

Clinically there is a shift in the symptomatology with nausea, vomiting and a pulsating character of the headaches becoming less often; otherwise the headache is more often located in the neck [75] or described as global or bilateral [76]. Amplification of the headache due to physical activity is less often reported and autonomic symptoms are less prominent or even missing [77]. In a small investigation in subjects (64 to 94 years old) living in a nursing home we found that most of the reported headaches could be classified as probable syndromes since specific features were not present, even when the subjects had had typical migraine in their youth. Acute medications, including triptans, seems to influence the attacks better than in younger patients [78, 79]. For 2/3 of the patients the attack frequency decreases with age [16] and also the intensity decreases [79]. In females about 20% of migraine patients lose their attacks per 10 years of life after the start of the menopause [16]. Aura symptoms with or without accompanying headache seem to occur more often in the elderly; in the group of 18–29-year-olds about 15.2% have auras compared to 41% of the patients aged 70 years and older [13, 77, 78]. As was also seen in middle-aged patients, the proportion of all migraine patients with frequent attacks is relatively higher in the elderly than in the other age groups [13].

As a physiological reason for this change in migraine representation in the elderly some authors propose a change in the reactivity of the cerebral blood vessels, since the dilatation of the intracranial vessels due to acetazolamide is less than that in younger patients [80]. This is somewhat supported by the finding that levels of VIP, SP, NPY, and CGRP, measured by immunofluorescence staining, in the middle cerebral artery decline with age [81]. The natural ageing of the brain causes changes in brain size, vasculature, and cognition, which range from a molecular level to morphology [82]. Whether trigemino-thalamic pathways and other areas involved in migraine pathophysiology undergo degenerative or connectivity changes during ageing has yet to be investigated. There is clear evidence that the autonomic nervous system, which is also part of the migraine pathophysiology, shows a significant decline with increasing age [83], which may explain the reduced autonomic symptoms in the elderly. A single study identified reduced gray matter volume in the secondary somatosensory cortex of elder migraineurs [84], while others studies demonstrated cortical thickening in younger patients [85, 86]. Reduced frontal cortex volume in the aged brain has been associated with cognitive changes; however, whether head pain perception is altered has not been investigated. In general, studies have shown an increased volume of white matter hyper intensities in elder migraine patients, while migraine with aura was found to be associated with brain infarcts, in the absence of any cognitive impairment [87, 88]. A study by Wen and colleagues even suggests that middle-aged and elderly migraine patients display better cognitive performance [89].

### Tension-type headache

#### Prevalence

The prevalence of tension-type headache is overall more variable than that of migraine. One reason for this may be that in some patients the characteristics of headache change over time and the diagnosis of tension-type headache has to be changed. In a review about global headache prevalence, Stover et al. reported on an average life-time prevalence of tension-type headache (all ages) of about 46% with large variability and the highest prevalence rates in Europe [90]. The prevalence in children was only slightly lower (31%) [90]. Otherwise the self-reported prevalence of tension-type headache in a Danish twin study was 86% (females slightly higher than males) and after the age of 39 years the prevalence declined for both sexes [91, 92]. This decline is less than that seen in migraine with increasing age [16]. For the group of 55–94-year-old subjects, the 12-month prevalence was 35.8% and 2.1% had chronic tension-type headache [16]. Females are more affected than males [93]. In the very old (> 70 years) the headache frequency seems to decrease again [94].

The prevalence for children is significantly less and in the global studies it was 31% (range: 10–72%) [90], with chronic tension-type headache nearly absent in the age group below 15 years [92].

#### Clinical symptoms in children

The symptoms bilateral location of the headache, mild intensity, pressing character, and no aggravation due to physical activity best differentiate tension-type headache from migraine in children [95]. In general, the symptoms of tension-type headache are not that different from those in adults. As seen in migraine, the duration of the attacks can be shorter and more variable than in adults [96]. There is no indication up to now that episodic syndromes [29] as described above are related to tension-type headache.

#### Clinical symptoms in adults

The problem with the classification of tension-type headache is that the criteria are relatively unspecific, which explains why several other headache syndromes, especially secondary headaches, can show a similar clinical manifestation. Tension-type headache has been called the featureless headache. The differentiation from secondary headaches is therefore sometimes difficult. In general the headache is described as bilateral, pressing or dull in quality and mild to maximally moderate in intensity. Interestingly there are an increasing number of reports which show that associated symptoms (osmophobia [97]) or co-morbid disorders (depression [98]) are quite similar to those described in migraine.

#### Clinical symptoms in the elderly

The clinical symptoms of tension-type headache do not fundamentally change with age. There is also no difference in the headache days between females and males [16]. A problem in all epidemiological studies concerning tension-type headache in the elderly is that due to the unspecific symptoms the percentage of patients with a secondary headache misdiagnosed as tension-type headache may increase [91]. The pathophysiological concept of tension-type headache in the elderly does not differ from that in younger patients and an increasing sensitization of central trigeminal nociceptive areas is discussed as the basic mechanism [99].

The pathophysiology of TTH has not been investigated in detail and no particular neural structures have been identified as being involved [100]. Certainly, such a pain in the region of the head demands activation of the ascending trigemino-thalamic pathway. Some studies suggest a role for peripheral sensitization of primary trigeminal afferents in the development of episodic TTH, potentially including muscle tenderness and inflammatory mediators [101]. Central sensitization that involves second-order neurons in the trigemino-cervical complex has been proposed for the development of chronic TTH [102], even in children suffering with TTH [103]. Chen et al. suggested differential cortical excitability in TTH patients compared to control subjects and migraine patients [104]. Gray matter changes as seen in TTH are reported only for middle-aged patients with no studies investigating this in children or the elderly [105, 106]. Otherwise, factors influencing neuronal structures in order to develop the reported age-dependent differences during the lifespan of TTH might be similar to those described above for migraine. For years there has been discussion as to whether tension-type headache and migraine are distinct entities or two poles of one disorder. Recently, a study concerning the comorbidity of migraine and tension-type headaches in twins concludes that there both have partially shared etiologies [107].

### Cluster headache

#### Prevalence

In a Swedish twin study the estimated life-time prevalence was 151/100000 and the male-to-female ratio 4.8 [108]. In the last years there seems to have been a decrease in the male preponderance [108]. Other studies, such as an epidemiological study by the German Headache Society (0.15%; 12-month prevalence) and an Italian study (0.28%; lifetime prevalence), reported a comparable prevalence [109, 110]. For episodic cluster headache, the onset was around the 20s for both sexes [111, 112], only the onset for females with chronic cluster headache seems to be evenly distributed between 10 and 69 years. Some studies see a peak incidence for males in the age group 40–49 years and females in the age group 60–69 years [79]. There are single case reports or case series about the first manifestation in the very young and very old patients. In a series of 11 children from a pediatric headache center the mean age of onset was 10 years (range 5–16 years) [113]. In another study focusing on patients younger than 13 years there was no male preponderance [114]. In contrast, the oldest patient with newly diagnosed cluster headache was 89 years old [115].

#### Clinical symptoms in children

So far the clinical presentation described for cluster headache in children seems to be very similar to that in adults. The mean duration of the attacks was 86 min and the daily attack frequency 1–4 attacks per day. All the children showed strictly unilateral pain around the orbit and autonomic symptoms such as lacrimation, ptosis, rhinorrhea, and conjunctival injection [113, 116]. In another study 35 patients with an onset before 18 years of age were described [117]. In the follow-up there was a tendency that the frequency and duration of the episodes increased, as did the attack frequency during the episodes [117]. In patients with pediatric onset of cluster headache, females tended to have more often a chronic course and in males there was a higher attack frequency and longer duration [114]. As seen in adults, steroids were also beneficial in children with cluster headache [113].

#### Clinical symptoms in adults

In a Chinese survey most often the pain was located temporally or retro-orbitally, lacrimation was the most frequently reported autonomic sign and about 40% also complained about photo- and phonophobia during the attacks [112]. No typical aura symptoms were seen. Concerning the frequency of bouts, about 40% had less than 1 bout per year and 40% 1–2 per year. The episodes occurred more often outside the summer time. The main duration of an untreated attack was 1.5 h [112]. The symptoms in the Chinese survey correspond well with reports from other regions [118, 119]. In our patient group (*n* = 105) we also found quite comparable clinical symptoms (personal observation). There is a high rate of active smokers amongst cluster headache patients (70%) [118]. Age of onset of the cluster headache probably has some implications for the clinical presentation. Patients with an onset after the age of 40 have a lower number of autonomic symptoms, especially conjunctival injection and nasal congestion [120]. The clinical phenotype is quite similar for female and male patients, the only difference might be that the attacks in males occur earlier in the evening [121]. There are no case series describing episodic syndromes, as have been done in migraine, but interestingly there is one case report which describes episodes of nocturnal awakening without headache or other autonomic signs some weeks before a new cluster bout in a patient with episodic cluster headache [122].

#### Clinical symptoms in the elderly

There are no reports in the literature dealing with the clinical presentation of cluster headache in the elderly. The case reports about late-onset cluster headache do not suggest a dramatic change in the clinical presentation. Some patients though report fewer and less severe attacks, however this has not been studied systematically.

Concerning the pathophysiology, most studies link cluster headache to activity of the hypothalamus [123]. Beside the imaging results, the circadian rhythm of the attacks and the annual rhythm are also in agreement with a primary involvement of hypothalamic structures. The hypothalamus is implicated as the activator of anatomical connections between the hypothalamus and the trigemino-vascular system, as well as the parasympathetic nervous system, giving rise to both pain and autonomic symptoms in the attack [124–126]. A change in the reactivity of the cerebral and extra-cerebral blood vessels, and potentially natural brain ageing that may influence neural connectivity may possibly account for any changes in the severity and frequency of cluster headaches during the lifespan.

### Other primary headaches

Concerning the other primary headaches (group 4 IHS), no adequate literature on the influence of age on the clinical presentation is available. One exception is hypnic headache which nearly exclusively occurs in subjects older than 50 years. Typically, the patients report on attacks of a bilateral dull moderate headache, which occurs during sleep (also during day-time sleep). The patients awake from that pain, no autonomic signs exist and the headache has an average duration of 15 min to 2 h. No differences between females and males are reported [127] and there are no reports on the clinical representation in the very old. Currently, there is no explanation for the age-dependent occurrence of hypnic headache.

## Discussion

Summarizing the main points about the prevalence and clinical presentation of primary headaches during lifespan the following points can be made:

1) Primary headache starts in a significant number of patients around the age of 5 years and has its maximal prevalence at around 20–40 years of life. The prevalence of primary headaches declines with older age but the percentage of patients with a high headache-day frequency is relatively higher in the elderly. Beside the primary headaches, the prevalence of secondary headaches increases with age [76].

2) The clinical presentation of tension-type headache is relatively similar for the different age groups with the exception of the significantly shorter attack duration in children. Otherwise, the clinical symptoms of migraine show a much larger variation with age. In children, the attacks are much shorter, the headache is often bilateral and some migraine-related syndromes nearly exclusively occur only in infancy and childhood. On the other hand, with increasing age migraine patients report nausea and vomiting less often and the headache becomes featureless, so that differentiation from tension-type headache becomes more difficult.

3) In the studies on cluster headache published so far, the prevalence was highest for the age group 20 to 40 years and the clinical presentation is quite similar across the different age groups, probably with the exception that for patients with a later onset the autonomic features are less prevalent.

In the following, we discuss possible reasons for, as well as consequences of, this change in the presentation of primary headaches during lifespan. Furthermore, we will discuss possible neuronal pathways, which may be responsible for the observed clinical symptoms.

One general feature, especially in migraine and less also in cluster headache, seems to be a decrease in autonomic symptoms during aging. The classical autonomic symptoms are – besides tearing and rhinorrhea – nausea and vomiting, all symptoms associated with increased parasympathetic activity. In the literature, there are a number of publications, which report on aberrations in autonomic functions in migraine patients. Unfortunately, no clear hypothesis can be drawn from these findings. Some describe increased sympathetic activity characterized by a peripheral circulation aberration probably of vasoconstrictive nature [128]. This is in contrast to the finding of lower sympathetic vasomotor activity in the supine position in adolescent migraineurs [129], which may explain the increased life-time prevalence of syncope and orthostatic intolerance in migraine patients [130]. Another study based on analysis of the electrocardiogram during sleep described reduced parasympathetic activity with sympathetic predominance [131]. In our own study we saw an exaggerated parasympathetic response to the pupillary reflex after a strong vegetative stimulus (cold water) [132] and a tendency to less parasympathetic and simultaneously more sympathetic activation in the control of the heart rate after mental stress or paced breathing [133]. One possible explanation for these different findings may be that there is a differential effect of migraine on the autonomic control of cranial autonomic reflexes and the cardiovascular system. No studies in which autonomic function was assessed in migraine patients in a longitudinal study or in different age groups have been published. Such a study would help to answer the question of whether the change in the reactivity of the autonomic system during life could be a reason for the decline in the prevalence of the autonomic symptoms during ageing and also why migraine symptoms in very young children are not as typical as in adolescents. The autonomic nervous system does typically show some changes over lifespan. In infancy (0–12 months) the cardiovascular response is more instable and variable and, in parallel to the development of a stable sleep architecture, the cardiovascular autonomic control becomes more stable during maturation [134]. Thus, it is not surprising that no differences to controls could be found in heart rate variability and the sympathetic skin response in studies with older children with migraine (9 to 17 years) [135]. There is some evidence that the cerebral control of autonomic cardiovascular control involves areas in the frontal lobe such as the anterior cingulate cortex, medial prefrontal cortex, insular cortex, thalamus, amygdala, and regions of the cerebellum [136]. These areas may combine in a feedback loop and influence the autonomic reflexes top-down [136]. The frontal cortex is one of the brain areas which mature late in infancy and frontal functions may not completely be established in the first years of life [137]. It is unclear if this immature control of the cortical control of autonomic functions is somewhat related to the time of migraine onset as well as clinical symptoms in children. On the other hand, there is clear evidence that there is also an age-related change in the autonomic nervous system [83, 138]. With regard to the cardiovascular control, the sympathetic influence increases with age and the parasympathetic influence decreases [139, 140]; in contrast, the sympathetic and the parasympathetic influence on the pupillary reaction are diminished [138, 141]. This diminished autonomic responsiveness in the elderly leads also to a diminished autonomic response to pain in the aged population [142]. It is not very clear if that is also true for the cranial autonomic nerves. It has not yet been investigated whether this change in the autonomic nerve system is related to the change in clinical symptoms during lifespan, but it is an interesting hypothesis, which may be the target of future studies. The influence of the autonomic system on primary headache is also demonstrated by the beneficial effect of non-invasive stimulation of vagal afferences in the prophylaxis of chronic cluster headache [143], the abortion of acute cluster attacks in episodic cluster headache [144] as well as in migraine attacks [145]. That stimulation of the cranial parasympathetic system may be helpful, is also supported by the observation that kinetic stimulation of the nasal concha, a strong parasympathetic stimulus, is able to abort migraine attacks [146], and electrical stimulation of the sensory representation of the vagal nerve in the ear conch is effective in the prophylaxis of chronic migraine [147]. Interestingly, internal vagal stimulation, as occurs during vomiting, is also able to abort a migraine attack in some patients [148], which may indicate that parasympathetic activation can abort the migraine attack.

A typical clinical feature of migraine attacks in children is the significantly shorter duration and the appearance of migraine-related disorders. The shorter duration can be theoretically explained by an earlier termination of the trigeminal activation due to the descending nociceptive system or a smaller activation of the trigeminal autonomic pathway or a stronger activation of parasympathetic pathways which are involved in the termination of headache attacks, as seen in experimental vagal activation. Most authors describe the descending anti-nociceptive system as hypoactive or even absent in infants [5]; therefore it seems unlikely that the descending inhibitory pathways are responsible for the shorter duration of the migraine attacks in childhood. On the other hand, autonomic symptoms are quite common in migraine attacks in children and there might even be a correlation between the attack frequency and the presence of autonomic signs [149]. Thus, some authors argue that the parasympathetic activation facilitates trigemino-vascular activation in migraine [149]. Concerning the above-cited results that external or internal vagal activation can also help to terminate migraine attacks, one can also discuss if the strong parasympathetic activation in childhood migraine also helps to terminate the attack early. With regard to the different location of the pain and the more frequent bilateral location of the pain, no physiological explanation has been discussed so far.

The episodic syndromes in infancy, which often are precursor symptoms of a later migraine, would be best explained by a temporarily disturbed descending inhibition, especially, reduced inhibition of the vestibular system (benign paroxysmal vertigo), of the descending axial motor system (benign paroxysmal torticollis, spinal vestibular pathways), and of the area postrema (cyclic vomiting) or the vagal control of the intestinal tract (abdominal migraine). Also sleepwalking and night terrors can be explained by disturbed control/inhibition of the motor system during sleep [150]. In summary, the episodic syndromes in infancy related to migraine are characterized by temporary deficits in descending control of brainstem circuits. Such an episodic disturbance of descending inhibitory pathways is also suggested as the pathophysiological basis of migraine. Migraine attacks are characterized by a complex pattern of sequential clinical symptoms, starting with premonitory symptoms like craving, yawning etc., followed by the headache and aura phase, and finishing with a recovery phase [151]. This sequence of clinical symptoms shows a cycling episodic recurrence, although the frequency varies for each individual. Based on imaging results, there has been discussion that the primary driver for migraine attacks is in the hypothalamic-brainstem connectivity [152], as was shown in an fMRI study in which a daily fMRI scan with trigeminal activation in patients with spontaneous migraine attacks was done over a period of 1 month [152]. The involvement of the hypothalamus in migraine is also supported by the resting state activity and the connectivity of the hypothalamus which was achieved in 12 interictal migraine patients [153]. Interestingly, the hypothalamus showed multiple connections with a number of areas, such as the locus coereleus, parahippocampal gyrus, or the superior temporal gyrus, involved in the control of autonomic functions [153]. Hypothalamic stimulation influences the volume of the gasto-intestinal tract by inhibition of the excitatory tone of the vagal nerve [154]. These connections could explain episodic syndromes such as abdominal migraine and cyclic vomiting by alteration of the central pathways of the vagal nerve. Interestingly, there are also connections from the lateral hypothalamus to the medial vestibular nucleus, a subnucleus of the vestibular complex. This input is generated from hypocretin cells and the medial vestibular nucleus projects to the arousal- and sleep-related areas in the brainstem [155]. The function of these pathways is not known in detail, but they would be suitable to be involved in the syndrome of benign paroxysmal vertigo, vestibular migraine, and also parasomnias. The lateral hypothalamus is also connected with the dorsal vagal complex by ghrelin-positive projections which may have an influence on emesis and nausea [156]. A completely different explanation for the episodic nystagmus and vertigo syndromes in children could be the occurrence of a paroxysmal spreading acidification and depression (SAD) in the cerebellum, a physiological reaction which could be shown in the cerebellum of rats and which was related to episodic ataxia type 1 (EA1), a syndrome caused by a mutation in the voltage-gated potassium channel with episodic and transient disturbance of the cerebellar function lasting a relatively short time. The disorder responds to acetazolamide which may be also beneficial in migraine with hemiparetic aura [157]. Up to now there is no proof that such a SAD can also be evoked in human cerebellum, but the paroxysmal short-lasting attacks of ataxia in EA1 support the idea. Recently, Mehnert and May did show a possible involvement of cerebellar neuronal circuits in migraine pathophysiology [158]. Migraineurs with an allodynia in the attacks showed a changed functional connectivity to the descending pain modulating system, as well as, to cerebellar, frontal and temporal regions [159]. Thus, cerebellar activity could influence migraine as well as vestibular and motor function. Interestingly, the cerebellum does show a strong change in activity during ageing with less activity in the senium compared to younger age [160]. There is experimental evidence that afferent vagal input to the caudal trigeminal nucleus is able to inhibit nociceptive input there [161]. Beside the vagal-trigeminal pathways, there are also reciprocal vestibular-trigeminal and vestibular-vagal connections [162, 163]. The role of these neuronal pathways in the development of vestibular symptoms during migraine attacks and paroxysmal episodic vertigo in childhood has not been investigated so far.

Concerning the change of clinical presentation with age, migraine is clearly distinguished from tension-type headache, which may be an argument to see tension-type headache as its own clinical entity. Otherwise, some co-morbidities, such as depression, sleep disorders, or restless leg syndrome, are similar for both primary headache syndromes. Thus, it is also possible that there is a gradual rather than a general difference in the hypothalamic connectivity to autonomic centres in the brainstem between both headache syndromes. Cluster headache pathophysiology is obviously related to the hypothalamic area, as is evident due to the strong chrono-biological dependence of the attacks, as well as, the changed functional connectivity outside and during a bout [164]. Furthermore, it is known that deep brain stimulation of hypothalamic areas successfully reduces the attack frequency in otherwise untreatable chronic cluster headache [165]. The autonomic activation in cluster headache is more intense than in migraine, although parasympathetic activation can also be seen in migraine [166]. Otherwise, there seems to be no relation of cluster headache to episodic syndromes in childhood, which probably differentiates the hypothalamic role in cluster headache from that in migraine.

In summary, there are typical changes in the clinical representation of migraine during lifespan. In general, in children we see episodic syndromes or sleep disorders more often than in adulthood. In the elderly the autonomic symptoms are less prominent or even absent. In contrast, there are no obvious changes in tension-type headache during ageing and in cluster headache there may also be a tendency to attenuation of autonomic symptoms.

**Fig. 1:**
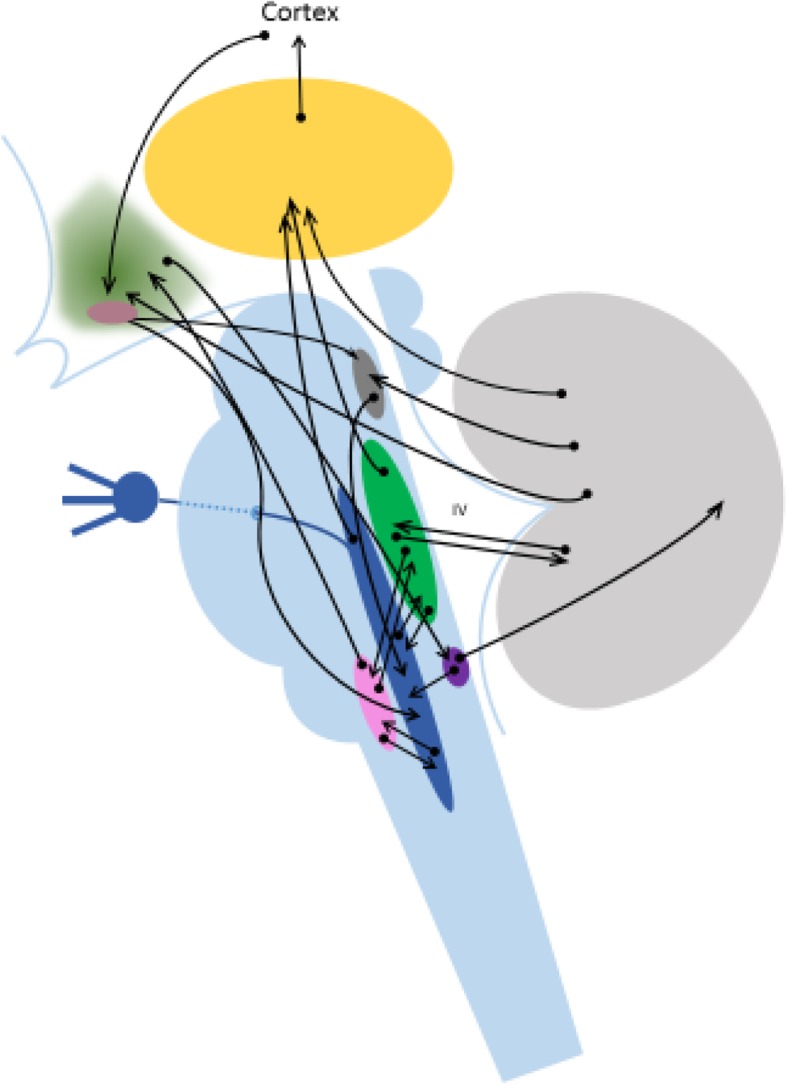
Schematic and incomplete drawing of hypothalmic and cerebellar pathways involved in the modulation of migraine and probably also of periodic syndromes [136, 155, 162, 163, 167–170]. The pathways are not differentiated in excitatory or functionally inhibitory pathways (e.g. the vagal connections to the spinal trigeminal nucleus are functionally inhibitory as it is also shown for the cerebellar pathways to the vestibular nucleus). green = hypothalamus, yellow = thalamus, grey = cerebellum; green = vestibular complex, blue = sensory trigeminal nucleus, pink = area postrema, rose = nucleus ambiguous (as part of the vagal complex)

A possible explanation is a change in the connectivity of hypothalamic areas or cerebellar areas to different autonomic control centres during ageing in migraine more than in cluster headache (Fig. 1 and Table 2).

**Table 2:** Clinical differences in primary headaches during lifespan

Phase of life/headache type	Migraine	Tension-type headache	Cluster headache
Childhood			
Gender (female/male)	Nearly 1:1(f:m)	Probably 1:1(f:m)	Probably 1:1
Attack duration	Less than 2 h	30 min to 2–4 h	15 min to 2 h
Pain characteristic	More often dull and on both sides	Dull, less intensity	Temporal, orbital, stabbing, high intensity
Autonomic symptoms	Vomiting, pallor, abdominal complaints often present	Absent	Typically present
Paroxysmal syndromes	Vertigo, vomiting, torticollis,	Absent	Absent
Adulthood			
Gender	2.5–3/1(f:m)	5:4 (f:m)	1:2.5–4 (f:m)
Attack duration	4 to 72 h	30 min to days	15 min to 3 h
Pain characteristic	Pulsating, hemicranial	Dull, less intensity	Temporal, orbital, stabbing, high intensity
Autonomic symptoms	Less often	Absent	Typically present
Paroxysmal syndromes	Rarely (cyclic vomiting)	Absent	Absent
Elderly			
Gender	2:1 (f:m), prevalence decreasing	5:4 (f:m), prevalence less decreasing	1:2.5–4 (f:m), prevalence mostly stable
Attack duration	4 to 72 h	30 min to days	15 min to 3 h
Pain characteristic	More often dull and on both sides	Dull, less intensity	Temporal, orbital, stabbing, high intensity
Autonomic symptoms	Mostly absent	Absent	Less pronounced
Paroxysmal syndromes	Absent	Absent	Absent

## Conclusions

Headache symptoms change during lifespan, especially, in migraine.

- In children the attacks are significantly shorter and episodic syndromes such as migraine equivalents can be observed. Autonomic symptoms are often prominent.
- In the elderly autonomic symptoms are less prominent and the headache becomes more featureless.

## Abbreviations

f: female
h: hours
m: male
min: minutes
