# Correction to: Primary headaches during lifespan

**DOI:** 10.1186/s10194-019-1025-9

**Published:** 2019-06-19

**Authors:** Andreas Straube, Anna P. Andreou

**Affiliations:** 1 Department of Neurology, University Hospital LMU, Ludwig-Maximilians-University, 81377 Munich, Germany; 2 Headache Research, Wolfson CARD, Institute of Psychiatry, Psychology and Neuroscience, King’s College London, London, UK; 3 The Headache Centre, Guy’s and St Thomas’, NHS Foundation Trust, London, UK

**Correction to: J Headache Pain**

**https://doi.org/10.1186/s10194-019-0985-0**

Following publication of the original article [1], we have been notified that one of the author names was incompletely presented. The correction can be seen below.

- Incomplete author name:
  Last Name: Andreou
  First Name: Anna
- Complete author name:
  Last Name: Andreou
  First Name: Anna P.
